# Supplementary material for: Assessment of time-related deficits in older adults: a scoping review protocol
Source: BMJ Open. 2021 Sep 24;11(9):e050521. doi: 10.1136/bmjopen-2021-050521 (PMC8475136; doi:10.1136/bmjopen-2021-050521)
Supplement: Supplementary data [file bmjopen-2021-050521supp001.pdf]

Assessment of time-related deficits in older adults: A scoping review protocol

Supplementary material. Example of search strategy for PubMed

(("SCREENING"[Title/Abstract] OR "ASSESSMENT"[Title/Abstract] OR "assess*"[All Fields] OR "TOOL"[Title/Abstract] OR "SCALE"[Title/Abstract] OR "INSTRUMENT"[Title/Abstract] OR "MONITOR"[Title/Abstract] OR "EVALUATE"[Title/Abstract] OR "IDENTIFY"[Title/Abstract] OR "TEST"[Title/Abstract]) AND ("TIME RELATED"[Title/Abstract] OR "TIME ORIENTATION"[Title/Abstract] OR "ROUTINE ACTIVITIES"[Title/Abstract] OR "COGNITIVE DEFICIT"[Title/Abstract]) AND ("humans"[MeSH Terms] AND 2000/01/01:2020/09/20[Date - Publication] AND ("middle aged"[MeSH Terms] OR "aged"[MeSH Terms]))) AND ((humans[Filter]) AND (2000/1/1:2020/9/20[pdat]) AND (middleaged[Filter] OR aged[Filter]))
