# Supplementary material for: Assessment of time-related deficits in older adults: a scoping review protocol
Source: BMJ Open. 2021 Sep 24;11(9):e050521. doi: 10.1136/bmjopen-2021-050521 (PMC8475136; doi:10.1136/bmjopen-2021-050521)
Supplement: Author's manuscript [file bmjopen-2021-050521.draft_revisions.pdf]

BMJ Open is committed to open peer review. As part of this commitment we make the peer review history of every article we publish publicly available.

When an article is published we post the peer reviewers' comments and the authors' responses online. We also post the versions of the paper that were used during peer review. These are the versions that the peer review comments apply to.

The versions of the paper that follow are the versions that were submitted during the peer review process. They are not the versions of record or the final published versions. They should not be cited or distributed as the published version of this manuscript.

BMJ Open is an open access journal and the full, final, typeset and author-corrected version of record of the manuscript is available on our site with no access controls, subscription charges or pay-per-view fees (http://bmjopen.bmj.com).

If you have any questions on BMJ Open's open peer review process please email info.bmjopen@bmj.com

BMJ Open

Assessment of time-related deficits in older adults: A scoping review

Journal:	*BMJ Open*
Manuscript ID	bmjopen-2021-050521
Article Type:	Protocol
Date Submitted by the Author:	22-Feb-2021
Complete List of Authors:	D'Souza, Sebestina Anita ; Manipal Academy of Higher Education, Department of Occupational Therapy, Centre for Studies on Healthy Aging, Manipal College of Health Professions<br>Ramachandran, Meena; McMaster University, Faculty of Health Sciences<br>Nishiura, Yuko; National Rehabilitation Center for Persons with Disabilities Research Institute, Department of Assistive Technology<br>VENKATESH, BHUMIKA; Manipal Academy of Higher Education, Public Health Evidence South Asia<br>Dahlberg, Lena; Dalarna University, School of Health and Social Studies; Karolinska Institutet & Stockholm University, Aging Research Center
Keywords:	Dementia < NEUROLOGY, Stroke < NEUROLOGY, Parkinson-s disease < NEUROLOGY, Delirium & cognitive disorders < PSYCHIATRY

SCHOLARONE™
Manuscripts

I, the Submitting Author has the right to grant and does grant on behalf of all authors of the Work (as defined in the below author licence), an exclusive licence and/or a non-exclusive licence for contributions from authors who are: i) UK Crown employees; ii) where BMJ has agreed a CC-BY licence shall apply, and/or iii) in accordance with the terms applicable for US Federal Government officers or employees acting as part of their official duties; on a worldwide, perpetual, irrevocable, royalty-free basis to BMJ Publishing Group Ltd ("BMJ") its licensees and where the relevant Journal is co-owned by BMJ to the co-owners of the Journal, to publish the Work in this journal and any other BMJ products and to exploit all rights, as set out in our licence.

The Submitting Author accepts and understands that any supply made under these terms is made by BMJ to the Submitting Author unless you are acting as an employee on behalf of your employer or a postgraduate student of an affiliated institution which is paying any applicable article publishing charge ("APC") for Open Access articles. Where the Submitting Author wishes to make the Work available on an Open Access basis (and intends to pay the relevant APC), the terms of reuse of such Open Access shall be governed by a Creative Commons licence – details of these licences and which Creative Commons licence will apply to this Work are set out in our licence referred to above.

Other than as permitted in any relevant BMJ Author's Self Archiving Policies, I confirm this Work has not been accepted for publication elsewhere, is not being considered for publication elsewhere and does not duplicate material already published. I confirm all authors consent to publication of this Work and authorise the granting of this licence.

For peer review only

Title of the article: Assessment of time-related deficits in older adults: A scoping review

Authors: Sebestina Anita Dsouza, Meena Ramachandran, Yuko Nishiura, Bhumika Tumkur Venkatesh, Lena Dahlberg

Full name, postal address and e-mail of the corresponding author

Dr Lena Dahlberg, School of Education, Health and Social Studies, Dalarna University, Sweden, and Aging Research Center, Karolinska Institutet & Stockholm University

Postal address: Lena Dahlberg, Dalarna University, 79188 Falun, Sweden.

Email: ldh@du.se

Full name, department, institution, city and country of all co-authors

- Dr Sebestina Anita Dsouza, Department of Occupational Therapy, Centre for Studies on Healthy Aging, Manipal College of Health Professions, Manipal Academy of Higher Education, Manipal, India- 576104.

  Email: sebestina.dsouza@manipal.edu

- Meena Ramachandran, Faculty of Health Sciences, McMaster University, Canada.

  Email: meena18790@gmail.com

- Yuko Nishiura, Research Institute, National Rehabilitation Center for Persons with Disabilities, Department of Assistive Technology, Japan.

  Email: nishiura-yuko@rehab.go.jp

- Bhumika T. V., Public Health Evidence South Asia, Prasanna School of Public Health, Manipal Academy of Higher Education, Manipal, India- 576104.

  Email: bhumika.tv@manipal.edu

For peer review only

Assessment of time-related deficits in older adults: A scoping review

Authors: Sebestina Anita Dsouza[1], Meena Ramachandran[2], Yuko Nishiura[3], Bhumika Tumkur Venkatesh.[4], Lena Dahlberg[5]

ABSTRACT

Introduction: People with cognitive impairments often have difficulties in managing their time for daily activities. In older adults with cognitive impairments such as dementia and stroke, these may present as disorientation, poor time awareness, time perception, daily time management, etc. Time-related deficits and associated behaviors impede independent living and increase caregiver strain. Several interventions are being investigated to help people with cognitive impairments orient and navigate time and do their daily activities. The provision of interventions requires the use of sound assessment tools. However, it is not clear how time-related concepts are specifically evaluated in practice, what are the available assessments and how these assessments should be selected.

Method and Analysis: We will search the following databases: PubMed, CINAHL, SCOPUS, Web of Science, PsychINFO. Two reviewers will independently screen identified studied for inclusion against the selection criteria and then review the full text of the selected studies. We will extract bibliographic data, study design and setting, and details of assessments used for evaluation of time-related concepts including format, mode and duration of administration, psychometric properties, etc. The identified assessments will be mapped with regard to the time-related concepts being evaluated and described using narrative synthesis.

Ethics and dissemination: As secondary data analysis, ethics approval is not required for this scoping review.

Registration: This protocol is registered with the Open Science Framework (https://osf.io/4ptgy/).

For peer review only

Strengths and limitations of this study

- The review will be comprehensive as we will be screening all studies published till the completion of our search.
- The protocol involves a rigorous methodological framework recommended by JBI.
- We may miss studies published in languages other than English and not available in databases included in this protocol.

For peer review only

Keywords: older adults, time-related cognitive functions, daily time management

INTRODUCTION

The ageing population is increasing worldwide and is expected to double by 2050.[1-2] While there are various health problems that affect older adults, neurological disorders are the most prevalent and debilitating. The disability-adjusted life years due to these disorders is significant and is expected to increase with the ageing population.[3] Cognitive impairments are a common clinical feature of several neurological disorders.

Among neurological disorders, dementia in older adults is a salient concern.[4] Dementia is a syndrome that has been described as a global epidemic, as the incidence and prevalence of dementia is increasing exponentially, especially in low and middle-income countries.[5-6] Besides dementia, cognitive impairments are common in an array of neurological conditions such as stroke, epilepsy, traumatic brain injury, Parkinson's disease, etc., and can range from subclinical to severe impairment.[7-8] Cognitive impairments, even when mild, are known to impact quality of life [7, 9] and participation[10], and contribute significantly to caregiver burden.[11]

Cognitive impairments include deficits in attention, memory, executive functions, learning capacity, etc., that impacts activity participation.[10] Participation in daily activities

also requires cognitive functions that are specifically related to understanding and managing time. These time-related cognitive functions help in planning and coordinating various daily occupations into patterns of time forming routine.[12] Several terms have been used to describe time-related abilities and cognitive functions. For convenience, in the present study, these are collectively referred to as time-related concepts and include time-related cognitive functions and daily time management abilities.

The International Classification of Functioning, Disability and Health (ICF) describes several time-related cognitive functions under the domain of body functions.[13] These include orientation to time (b1140) and experience of time (b1802) and also time management (b1642), the latter as a component of executive functions (b164). To facilitate better understanding, Janeslatt has organised the various time-related cognitive functions in a three-level hierarchy within a single construct of 'time processing ability' (TPA).[14-15] Within this construct, time perception refers to the experience of time and is at the lowest level of this hierarchy. It entails the ability to know how time passes and how much time is required for different activities. Time orientation is at the next level of TPA and involves awareness of time such as the time of the day, date, day of the week, month and year. Finally, time management is at the highest level of TPA that refers to the ability to plan and order daily activities chronologically and to know how much time to allocate for these activities.[14, 16] Time management is a cognitive function under TPA and is distinct from the concept of daily time management.

In the ICF, the concept of daily time management is referred to as managing one's time (d2305), i.e., time required to complete one's usual or specific activities in daily life. [17] The impact of time is further shown in the ICF domain of activity and participation which has, in all, seven components related to the use of time in daily activities.[13] One example is 'carrying out daily routine (d230)' under general tasks and demands. This includes managing daily routine (d2301), completing daily routine (d2302), managing one's own activity level (d2303),

For peer review only

carrying out specified (d2308) and unspecified (d2309) daily routines. Time is embedded in this complex component and cuts across many other components in the domain of activity and participation.

People with cognitive impairments are known to have difficulties in managing time for their daily activities.[18-20] Difficulties in time-related cognitive functions could present in different ways in different populations of older adults. Persons with dementia are known to have disorientation to the time, day and date, require more time for daily activities, difficulty in keeping track of past events, planning for future events, difficulty knowing, remembering and planning "when" and difficulty in judging the duration of different activities and situations.[21] Haj and Kapogiannis have described time distortions in dementia due to deficits in memory and time perception.[22] Difficulties in time estimation have been attributed to deficits in central executive function[23] and distortions in different components of the internal clock.[24] Requena-Komuro[25] have reported altered time awareness that contributes to abnormal behaviours in dementia syndromes such as clockwatching, reliving past events and temporal inflexibility and the Godot syndrome, i.e., anxiety symptoms regarding upcoming events by repeatedly asking questions. Difficulties with time estimation have been reported in individuals with mild cognitive impairments[24, 26] and also in older adults without cognitive impairments.[27]

Time-related deficits and associated behaviours affect the ability to do daily activities independently and add considerably to caregiver strain and distress. The ability to manage one's time effectively is an essential factor influencing a person's self-efficacy.[28-29] With increasing understanding of the importance of time-related abilities on performance of daily activities, several interventions are being investigated to help people with cognitive impairments orient and navigate time and do their daily activities. These include reality orientation,[30] high-tech assistive devices such as time aids, automatic calendars,[21, 31, 32]

For peer review only

electronic devices,[39] mobile-based applications,[33] and low-tech assistive devices such as paper-based calendars,[34[routine organizers[35] and training to improve organizational skills and habits.[36-37]

The provision of interventions requires the use of sound assessments to screen, evaluate or identify the nature and severity of time-related cognitive deficits and their impact on performance of daily activities. They are also essential to monitor and document changes with intervention or disease progression. Assessments have also been used by occupational therapists to design interventions for children and youth having difficulties with time-related concepts.[38, 40] However, it is not clear how time-related cognitive functions and daily time management of older adults are evaluated in practice, what are the available assessments and how these assessments should be selected.

A preliminary search for existing reviews on this topic was done in the following databases: JBI database of Systematic Reviews and Implementation Reports, Cochrane Database of Systematic Reviews, Cumulative Index to Nursing and Allied Health Literature (CINAHL), PROSPERO, and PubMed. We did not find any review addressing assessment of time-related concepts in older adults. The proposed scoping review intends to address this lacuna by systematically mapping research done in this area.

Research question

What are the available tools to assess time-related cognitive functions and daily time management in older adults?

Aim

The aim of this proposed scoping review is to investigate assessments for time-related cognitive functions and daily time management in older adults with and without cognitive impairments.

For peer review only

Objectives

- To map the assessments tools available for older adults with respect to time-related concepts.
- To describe the characteristics of available assessment tools including, purpose, type and format of the test, study populations, psychometric properties and feasibility.

METHODS

The protocol was drafted using the Joanna Briggs Institute (JBI) Reviewer's Manual.[41] The protocol is registered with the Open Science Framework (https://osf.io/4ptgy/). The review will involve the following steps:

1. **Identification of relevant studies**: The selection criteria and search strategy to identify relevant studies are as follows:

   a. Selection criteria**:**

   - Articles with older adults as participants, i.e., people aged 60 years or older OR groups with an average age of 60 years or older,[5] with and without cognitive impairments will be included. Articles that have older participants along with other age groups will also be included.
   - Primary research articles (quantitative and mixed method studies) that discuss assessment tools for one or more time-related concepts described in the introduction, either as a separate/specific test or as a component of another test (such as a cognitive assessment), if results of that component are presented separately. This includes:
     - Pre-post, quasi-experimental and experimental (randomized and non-randomized controlled trials) designs evaluating the effectiveness of interventions targeting time-related concepts (if the outcome measures used

For peer review only

include assessments of time-related concepts) and also observational studies investigating one or more time-related concepts, and studies developing or validating an instrument or assessment tool for one or more time-related concepts. Articles with experimental laboratory-based designs will be excluded.

- All peer-reviewed articles in English till date will be included. The language restriction to English is based on convenience and feasibility of the authors involved in the study group.

b. Search strategy: The keywords will be identified and search strategy will be constructed with involvement of all the authors. The search will be conducted in the following databases, PubMeD, CINAHL, SCOPUS, Web of Science, PsychINFO. First an initial limited search of at least two databases will be done such as PubMed and CINAHL. This will be followed by analysis of the text words contained in the title and abstract of the retrieved publications and the index terms used to describe the articles. A second search using all identified keywords and index terms will be undertaken across all included databases. The reference list of included publications will be searched for additional sources. The bibliographic information of included publications will be combined and stored using EndNote. The key terms for the search are summarized in Table 1. The search terms will be refined further if required. We plan to conduct the search between September and October 2020. A combination of descriptors (e.g. MESH terms) and keywords will be used with appropriate Boolean operators. The search strategy will be tailored to each database.

For peer review only

Table 1: Keywords for search

Keyword	Alternate keywords
Older adults with and without cognitive impairments	elderly, older, older age, aged, older adults, elderly persons, old, seniors, aging adults, older persons, geriatric, executive dysfunction, mild cognitive impairment, dementia, delirium, cognitive impairment, cognitive dysfunction, cognitive limitation, Alzheimer's, senile, psychiatric disorders, neurological disorders, stroke, Parkinson's,
Assessment tools	assessment tool, screening, tool, evaluation tool, diagnostic, psychometric properties, psychometrics, validity, reliability, test, prediction, predictive value, questionnaire, instrument,
Time-related concepts	time, time sense, time perception, time orientation, time awareness, time processing abilities, time management/managing time, routines, schedule, orientation, disorientation, time planning, executive functions, cognitive functions, cognition, cognitive abilities, judging time, time judgement, time organization, organizing time, task duration, activity duration, temporal, temporality, calendar.

2. **Selection of articles (relevance):** Two reviewers will independently review the title and abstract of the included identified publications against the selection criteria. They will be marked as 'potentially relevant' or 'potentially irrelevant'. Discrepancies, if any, will be resolved by discussion and consensus between the two reviewers. If not resolved, a third reviewer will be consulted. This will be pilot tested using the

For peer review only

framework proposed by JBI.[41] Full text publications will be retrieved for studies regarded as ‘potentially relevant’, and will be reviewed independently and in duplicate for inclusion against the selection criteria. All reviewers will use a pilot-tested screening form developed for this review.

3. **Data extraction:** Two reviewers will independently review the included articles using a data extraction form. The data extraction form will be adapted from JBI.[41] It will be piloted for approximately five articles. Modifications if any will be made and the remaining articles will be reviewed with the finalized data extraction form. The details to be extracted would include:

   a. Bibliometric data: title of the study, journal, year of publication, place (country) where the study was conducted.
   b. Study details: aim/purpose/research question, study design, study setting (institution, community, in-patient, etc.), study population (older adults with cognitive impairment, without cognitive impairment, etc.), mean age of participants, sample size.
   c. Details of time-related assessments used in the study: time-related concepts being assessed, purpose/type of assessment (screening, diagnostic), language, format (computer/paper-pencil), mode of administration (self-report/therapist-administered), cost of using the assessment tool (paid/free), time for the test, psychometric properties, etc.

4. **Collating, summarizing and reporting the results**: An overview of the included publications will be provided in a tabular format to summarize the various aspects such as study type, aim, study design, settings, participants and time-related cognitive

For peer review only

assessments used. As it is a scoping review, quality assessment of the identified articles will not be undertaken**.** The identified assessments will be mapped with regard to time-related concepts being assessed. A narrative synthesis will be carried out for test characteristics and to summarize the findings. Reporting will be done using the Preferred Reporting Items for Systematic reviews and Meta-Analyses extension for Scoping Reviews.[42]

DISCUSSION

The present protocol outlines a scoping review of peer-reviewed articles on assessments available for time-related concepts in older adults and to our knowledge, this is the first of its kind. This review will advance knowledge about assessing time-related concepts in older adults. This area is gaining importance due to its impact on function and participation of older adults and their caregivers. The review will inform the selection of appropriate instruments to evaluate different time-related concepts in older adults. It will guide clinical reasoning in planning interventions to support daily time management. The review will also identify gaps to be addressed in future research. If sufficient literature is identified, a future systematic review or meta-analysis may be warranted.

Ethics and dissemination

As a secondary analysis, ethics approval is not required for this scoping review. We plan to disseminate the results through peer-reviewed journals and conferences targeting health professionals working with older adults. Amendments to the protocol, if any, will be stated in any future publication based on the review presented in this protocol.

For peer review only

Author Affiliations

[1]Department of Occupational Therapy and Centre for Studies on Healthy Aging, Manipal College of Health Professions, Manipal Academy of Higher Education, Manipal, Karnataka, India

[2]Meena Ramachandran, Faculty of Health Sciences, McMaster University, Canada

[3]Yuko Nishiura, Research Institute, National Rehabilitation Center for Persons with Disabilities, Department of Assistive Technology, Japan

[4]Bhumika Tumkur Venkatesh, Public Health Evidence South Asia, Prasanna School of Public Health, Manipal Academy of Higher Education, Manipal, Karnataka, India

[5]Lena Dahlberg, School of Education, Health and Social Studies, Dalarna University, Sweden, and Aging Research Center, Karolinska Institutet & Stockholm University, Sweden

Acknowledgements: The authors would like to thank Dr. Gunnel Janeslätt, Researcher, Center for Clinical Research, Dalarna University, Dalarna and associated to Centre for Public Health and Caring Sciences, Disability and Habilitation, Uppsala University, Uppsala, Sweden for her valuable guidance and support to this scoping review protocol.

Authors' contributions: All authors have made substantive intellectual contributions to the development of this protocol. SAD conceived the idea of this research, followed by discussions with the other authors that contributed to finalising the research idea. All authors worked on the methodology of the scoping review. SAD drafted the protocol with inputs from MR, YN and BTV which was further reviewed and revised by LD.

For peer review only

Funding: This scoping review is part of an ongoing international research collaboration "Managing Time in Dementia" funded by the Indian Council of Medical Research (54/1/GER/Indo-Sweden/17-NCD-II), India; Swedish Research Council for Health, Working Life and Welfare, (FORTE 2017-00029), Sweden; and Japan Society for the Promotion of Science, Grant-in-Aid for Young Scientists (B) [16K16458], Japan.

Competing interests: None declared.

Patient and public involvement: Patients and/or the public were not involved in the design, or conduct, or reporting, or dissemination plans of this research.

Patient consent for publication: Not required.

For peer review only

References

1. World Health Organization. Ageing and Health, 2018. Available at: https://www.who.int/news-room/fact-sheets/detail/ageing-and-health (accessed 29 September 2020).
2. United Nations. World Population Ageing Highlights, 2017. Available at: https://www.un.org/en/development/desa/population/publications/pdf/ageing/WPA2017_Highlights.pdf (accessed 29 September 2020).
3. GBD 2016 Neurology Collaborators. Global, regional, and national burden of neurological disorders, 1990–2016: a systematic analysis for the Global Burden of Disease Study 2016. *Lancet Neurol* 2019;18:459–80. doi:https://doi.org/10.1016/S1474-4422(18)30499-X
4. Global Burden of Disease Collaborative Network, 2012. Global Burden of Disease Study 2010 (GBD 2010) Results by Cause 1990-2010. Seattle, United States: Institute for Health Metrics and Evaluation (IHME). doi:https://doi.org/10.6069/J86D-DZ43

5. World Health Organization. The epidemiology and impact of dementia: current state and future trends, 2015. Available at https://www.who.int/mental_health/neurology/dementia/dementia_thematicbrief_epidemiology.pdf (accessed 29 September 2020).

6. World Health Organization. Dementia Fact Sheet, 2020. Available at: www.who.int/mediacentre/factsheets/fs362/en/ (accessed 29 September 2020).

7. Mitchell AJ, Kemp S, Benito-León J, Reuber M. The influence of cognitive impairment on health-related quality of life in neurological disease. *Acta Neuropsychiatr* 2010;22:2-13 doi:10.1111/j.1601-5215.2009.00439.x

8. Danovska M, Stamenov B, Alexandrova M, Peychinska D. Post-stroke cognitive impairment - phenomenology and prognostic factors. *Journal of IMAB* 2012;18:290-297. doi:10.5272/jimab.2012183.290

9. Cullen B, Smith DJ, Deary IJ, et al. The 'cognitive footprint' of psychiatric and neurological conditions: cross-sectional study in the UK Biobank cohort. *Acta Psychiatr Scand* 2017;135:593-605. doi:10.1111/acps.1273

10. Rotenberg S, Maeir A, Dawson DR. Changes in activity participation among older adults with subjective cognitive decline or objective cognitive deficits. *Front Neurol* 2020;10:1393. doi:10.3389/fneur.2019.01393

11. Black CM, Ritchie CW, Khandker RK et al. Nonprofessional caregiver burden is associated with the severity of patients' cognitive impairment. *PLoS One* 2018;13:e0204110. doi:https://doi.org/10.1371/journal.pone.0204110

For peer review only

12. Rosenblum S, Regev N. Timing abilities among children with developmental coordination disorders (DCD) in comparison to children with typical development. *Res Dev Disabil* 2013;34:218-227. doi:https://doi.org/10.1016/j.ridd.2012.07.011

13. World Health Organization. International Classification of Functioning Disability and Health, 2001. Available at https://www.who.int/standards/classifications/international-classification-of-functioning-disability-and-health (accessed 4 December 2020)

14. Janeslätt G. Validity in assessing time processing ability, test equating of KaTid-Child and KaTid-Youth. *Child Care Health Dev* 2012;38:371-378. doi:https://doi.org/10.1111/j.1365-2214.2011.01249.x

15. Janeslätt G, Granlund M, Alderman I et al. Development of a new assessment of time processing ability for children, using Rasch analysis. *Child Care Health Dev* 2008;34:771-780. doi:10.1111/j.1365-2214.2008.00865.x

16. Janeslätt G, Kottorp A, Granlund M. Evaluating intervention with time aids in children with disabilities. *Scand J Occup Ther* 2014;21:181-190. doi:https://doi.org/10.3109/11038128.2013.870225

17. WHO. ICF Update Platform, 2021. Available from: https://extranet.who.int/icfrevision/nr/loginICF.aspx. (accessed 11 January, 2021)

18. Davies D, Stock S, Wehmeyer M. Enhancing independent time-management skills of individuals with mental retardation using a palmtop personal computer. *Ment Retard* 2002;40:358-65. doi:10.1352/0047-6765(2002)040<0358:EITMSO>2.0.CO;2.

19. Toner M , O'Donoghue T, Houghton S. Living in chaos and striving for control: How adults with Attention Deficit Hyperactivity Disorder deal with their disorder. *Intl J Disabil Dev Educ* 2006;53:247-261. doi: 10.1080/10349120600716190

For peer review only

20. Borson S. Cognition, Aging and Disabilities: Conceptual Issues. *Phys Med Rehabil Clin N Am* 2010;21:375-382. doi:10.1016/j.pmr.2010.01.001

21. Nygård L, Johannson M. The experience and management of temporality in five cases of dementia. *Scand J Occup Ther* 2001;8:85-95. doi:https://doi.org/10.1080/11038120120542

22. Haj EM, Kapogiannis D. Time distortions in Alzheimer's disease: a systematic review and theoretical integration. *NPJ Aging Mech Dis* 2016; 2:1-5. doi:10.1038/npjamd.2016.16;

23. Papagno C, Allegra A, Cardaci M. Time estimation in Alzheimer's disease and the role of the central executive. *Brain and Cognition* 2004;54:18-23. doi:10.1016/S0278-2626(03)00237-9

24. Caselli L, Iaboli L, Nicheli P. Time estimation in Alzheimer's disease patients. *Behav Brain Sci* 2009;5:1-10. doi:10.1186/1744-9081-5-32

25. Requena-Komuro MC, Marshall CR, Bond RL, et al. Altered Time Awareness in Dementia. *Front Neurol* 2020;11:1-12. doi:10.3389/fneur.2020.00291

26. Rueda AD, Schmitter-Edgecombe M. Time estimation abilities in mild cognitive impairment and Alzheimer's disease. *Neuropsychology* 2009;23(2):178–188. doi:10.1037/a0014289

27. Toglia J, Lahav O, Ari EB, et al. Adult age and cultural differences in performance on the Weekly Calendar Planning Activity (WCPA). *Am J Occup Ther* 2017;71(5):7105270010p1-8. doi:10.5014/ajot.2016.020073

28. Bembenutty H. Academic delay of gratification, self-efficacy, and time management among academically unprepared college students. *Psychol Rep* 2009;104:613-23. doi:10.2466/pr0.104.2.613-623

For peer review only

29. Hayat RA, Janeslätt G, White S et al. Time management skills in relation to general self-efficacy and parental sense of competence in individuals with and without cognitive disabilities. *Cogent Psychol 2019*;6:1. doi: 10.1080/23311908.2019.1655981

30. Camargo, CHF, Justus FF, Retzlaff G. The effectiveness of reality orientation in the treatment of Alzheimer's Disease. *Am J Alzheimers Dis Other Demen* 2015;30:527-532. doi: 10.1177/1533317514568004

31. Lindqvist E, Nygard L, Borrell L. Significant junctures on the way towards becoming a user of assistive technology in Alzheimer's disease. *Scand J Occup Ther* 2013;20:386-396. doi: 10.3109/11038128.2013.766761

32. Nishiura Y, Nihei M, Nakamura-Thomas H, et al. Effectiveness of using assistive technology for time orientation and memory, in older adults with or without dementia. *Disabil Rehabil Assist Technol* 2019;19:1-7. doi:10.1080/17483107.2019.1650299

33. Jamieson M, Cullen B, Lennon M, Brewster S, Evans J. Designing ApplTree: usable scheduling software for people with cognitive impairments. *Disabil Rehabil Assist Technol* 2020. doi:10.1080/17483107.2020.1785560.

34. Greenaway MC, Duncan NL, Smith GE. The memory support system for mild cognitive impairment: randomized trial of a cognitive rehabilitation intervention. *Int J Geriatr Psychiatry* 2013;28:402-409.

35. Assis LDO, Tirado MGA, Pertence AEDM, et al. Evaluation of cognitive technologies in geriatric rehabilitation: A case study pilot project. *Occup Ther Int* 2010;17:53–63.

36. Holmefur M, Lidström-Holmqvist K, Roshanay AH, Arvidsson P, White S, Janeslätt G. Pilot study of Let's Get Organized: A group intervention for improving time management. *Am J Occup Ther* 2019;73:7305205020. Available at: https://doi.org/10.5014/ajot.2019.032631

For peer review only

37. White S. Let's get organized: An intervention for persons with co-occurring disorders. *Psychiatr Serv* 2007 May;58(5). Available at: https://ps.psychiatryonline.org/doi/full/10.1176/ps.2007.58.5.713 (accessed 29 September 2020).

38. Wennberg B, Janeslätt G, Kjellberg A, Gustafsson PA. Effectiveness of time-related interventions in children with ADHD aged 9-15 years: a randomized controlled study. *Eur Child Adolesc Psychiatry* 2018;27:329-342. doi: 10.1007/s00787-017-1052-5.

39. Janeslätt G, Lindstedt H, Adolfsson P. Daily time management and influence of environmental factors on use of electronic planning devices in adults with mental disability. *Disabil Rehabil Assist Technol* 2014;10:371-377. doi: 10.3109/17483107.2014.917124.

40. Janeslätt G, Granlund M, Kottorp A. Measurement of time processing ability and daily time management in children with disabilities. *Disabil Health J* 2009;2:15-9. doi: 10.1016/j.dhjo.2008.09.002.

41. Peters MDJ, Godfrey C, McInemey P, et al. Scoping reviews. In: Aromataris E, Munn Z, eds. Joanna Briggs Institute Reviewer's Manual. Adelaide, Australia: Joanna Briggs Institute; 2020:407-454.

42. Tricco, AC, Lillie, E, Zarin, W, O'Brien, KK, Colquhoun, H, Levac, D, Moher, D, Peters, MD, Horsley, T, Weeks, L, Hempel, S et al. PRISMA extension for scoping reviews (PRISMA-ScR): checklist and explanation. *Ann Intern Med* 2018;169:467-473. doi:10.7326/M18-0850.

For peer review only

BMJ Open

Assessment of time-related deficits in older adults: A scoping review protocol

Journal:	*BMJ Open*
Manuscript ID	bmjopen-2021-050521.R1
Article Type:	Protocol
Date Submitted by the Author:	24-Aug-2021

Complete List of Authors:	Dsouza, Sebestina; Manipal Academy of Higher Education, Department of Occupational Therapy, Centre for Studies on Healthy Aging, Manipal College of Health Professions<br>Ramachandran, Meena; McMaster University, Faculty of Health Sciences<br>Nishiura, Yuko; National Rehabilitation Center for Persons with Disabilities Research Institute, Department of Assistive Technology<br>VENKATESH, BHUMIKA; Manipal Academy of Higher Education, Public Health Evidence South Asia<br>Dahlberg, Lena; Dalarna University, School of Health and Social Studies; Karolinska Institutet & Stockholm University, Aging Research Center

**Primary Subject Heading**:	Occupational and environmental medicine
Secondary Subject Heading:	Geriatric medicine
Keywords:	Dementia < NEUROLOGY, Stroke < NEUROLOGY, Parkinson-s disease < NEUROLOGY, Delirium & cognitive disorders < PSYCHIATRY

SCHOLARONE™
Manuscripts

I, the Submitting Author has the right to grant and does grant on behalf of all authors of the Work (as defined in the below author licence), an exclusive licence and/or a non-exclusive licence for contributions from authors who are: i) UK Crown employees; ii) where BMJ has agreed a CC-BY licence shall apply, and/or iii) in accordance with the terms applicable for US Federal Government officers or employees acting as part of their official duties; on a worldwide, perpetual, irrevocable, royalty-free basis to BMJ Publishing Group Ltd ("BMJ") its licensees and where the relevant Journal is co-owned by BMJ to the co-owners of the Journal, to publish the Work in this journal and any other BMJ products and to exploit all rights, as set out in our licence.

The Submitting Author accepts and understands that any supply made under these terms is made by BMJ to the Submitting Author unless you are acting as an employee on behalf of your employer or a postgraduate student of an affiliated institution which is paying any applicable article publishing charge ("APC") for Open Access articles. Where the Submitting Author wishes to make the Work available on an Open Access basis (and intends to pay the relevant APC), the terms of reuse of such Open Access shall be governed by a Creative Commons licence – details of these licences and which Creative Commons licence will apply to this Work are set out in our licence referred to above.

Other than as permitted in any relevant BMJ Author's Self Archiving Policies, I confirm this Work has not been accepted for publication elsewhere, is not being considered for publication elsewhere and does not duplicate material already published. I confirm all authors consent to publication of this Work and authorise the granting of this licence.

For peer review only

Title of the article: Assessment of time-related deficits in older adults: A scoping review protocol

Authors: Sebestina Anita Dsouza[1], Meena Ramachandran[2], Yuko Nishiura[3], Bhumika Tumkur Venkatesh.[4], Lena Dahlberg[5]

Full name, postal address and e-mail of the corresponding author

Dr Lena Dahlberg, School of Health and Welfare, Dalarna University, Sweden; and Aging Research Center, Karolinska Institutet & Stockholm University

Postal address: Lena Dahlberg, Dalarna University, 79188 Falun, Sweden.

Email: ldh@du.se

Full name, department, institution, city and country of all co-authors

- Dr Sebestina Anita Dsouza, Department of Occupational Therapy, Centre for Studies on Healthy Aging, Manipal College of Health Professions, Manipal Academy of Higher Education, Manipal, India- 576104.

  Email: sebestina.dsouza@manipal.edu

- Meena Ramachandran, Faculty of Health Sciences, McMaster University, Canada.

  Email: meena18790@gmail.com

- Yuko Nishiura, Research Institute, National Rehabilitation Center for Persons with Disabilities, Department of Assistive Technology, Japan.

  Email: nishiura-yuko@rehab.go.jp

- Bhumika T. V., Public Health Evidence South Asia, Prasanna School of Public Health, Manipal Academy of Higher Education, Manipal, India- 576104.

  Email: bhumika.tv@manipal.edu

For peer review only

For peer review only - http://bmjopen.bmj.com/site/about/guidelines.xhtml

Assessment of time-related deficits in older adults: A scoping review protocol

Authors: Sebestina Anita Dsouza[1], Meena Ramachandran[2], Yuko Nishiura[3], Bhumika Tumkur Venkatesh[4], Lena Dahlberg[5]

ABSTRACT

Introduction: People with cognitive impairments often have difficulties in managing their time for daily activities. In older adults with cognitive impairments such as dementia and stroke, these may present as disorientation, poor time awareness, time perception, daily time management, etc. Time-related deficits and associated behaviors impede independent living and add considerably to caregiver strain. Several interventions are being investigated to help people with cognitive impairments orient and navigate time and do their daily activities. The provision of interventions requires the use of sound assessment tools. However, it is not clear how time-related concepts are specifically evaluated in practice, what are the available assessments and how these assessments should be selected.

Method and Analysis: This protocol follows the Joanna Briggs Institute Reviewer's Manual (2020) for scoping reviews and is registered with the Open Science Framework (https://osf.io/4ptgy/). We will include the following databases: PubMed, CINAHL, SCOPUS, Web of Science, PsychINFO. Two reviewers will independently screen eligible studies for inclusion against the selection criteria and then review the full-text of the selected studies. We will extract the bibliographic data, study design and setting, and details of assessments used in the studies to evaluate time-related concepts including format, mode and duration of administration, psychometric properties, etc. The identified assessments will be mapped with regard to time-related concepts being evaluated and described using narrative synthesis.

For peer review only - http://bmjopen.bmj.com/site/about/guidelines.xhtml

Ethics and dissemination: As secondary data analysis, ethics approval is not required for this scoping review. We plan to disseminate the results through peer-reviewed journals and conferences targeting health professionals working with older adults.

Strengths and limitations of this study

- The review will be comprehensive as we will be screening all studies published till the completion of our search.
- The protocol involves a rigorous methodological framework recommended by JBI.
- We may miss studies published in languages other than English and not available in databases included in this protocol.

Keywords: older adults, time-related cognitive functions, daily time management

INTRODUCTION

The ageing population is increasing worldwide and is expected to double by 2050 [1-2]. While there are various health problems that affect older adults, neurological disorders are the most prevalent and debilitating. The disability-adjusted life years due to these disorders is significant and is expected to increase with the ageing population [3]. Cognitive impairments are a common clinical feature of several neurological disorders.

Among neurological disorders, dementia in older adults is a salient concern [4]. Dementia is a syndrome that has been described as a global epidemic, as the incidence and prevalence of dementia is increasing exponentially, especially in low and middle-income countries [5-6]. Besides dementia, cognitive impairments are common in an array of neurological conditions such as stroke, epilepsy, traumatic brain injury, Parkinson's disease, etc., and can range from subclinical to severe impairment [7-8]. Cognitive impairments, even

For peer review only - http://bmjopen.bmj.com/site/about/guidelines.xhtml

For peer review only

when mild, are known to impact quality of life [7, 9] and participation [10], and contribute significantly to caregiver burden [11].

Cognitive impairments include deficits in attention, memory, executive functions, learning capacity, etc., that impacts activity participation [10]. Thus, participation in daily activities also requires cognitive functions that are specifically related to time. These time-related cognitive functions help in planning and coordinating various daily occupations into patterns of time forming routine [12]. Several terms have been used to describe time-related abilities and cognitive functions. For convenience, in the present study, these are collectively referred to as time-related concepts and include time-related cognitive functions and daily time management abilities.

The International Classification of Functioning, Disability and Health (ICF) describes several time-related cognitive functions under the domain of body functions [13]. These include orientation to time (b1140) and experience of time (b1802) and also time management (b1642), the latter as a component of executive functions (b164). To facilitate better understanding, Janeslätt has organised the various time-related cognitive functions in a three-level hierarchy within a single construct of 'time processing ability' (TPA) [14-15]. Within this construct, time perception refers to the experience of time and is at the lowest level of this hierarchy. It entails the ability to know how time passes and the duration of different activities. Time orientation is at the next level of TPA and involves awareness of time such as the time of the day, date, day of the week, month and year. Finally, time management is at the highest level of TPA that refers to the ability to plan and order daily activities chronologically and to know how much time to allocate for these activities [14, 16]. Time management is a cognitive function under TPA and is distinct from the concept of daily time management.

Time is an integral and salient component in the performance of daily activities. This is well described in the activity and participation domain of the ICF which has, in all, seven

For peer review only - http://bmjopen.bmj.com/site/about/guidelines.xhtml

components related to the use of time in daily activities [13]. One example is 'carrying out daily routine (d230)' under general tasks and demands (d2). This includes managing daily routine (d2301), completing daily routine (d2302), managing one's own activity level (d2303), adapting to changes in daily routine (2304), managing one's time (d2305), carrying out specified (d2308) and unspecified (d2309) daily routines. This shows how time is embedded in this complex component of carrying out one's daily routine and cuts across other components in the domain of activity and participation. Managing one's time (d2305) is described as 'managing the time required to complete usual or specific activities, actions and behaviours appropriately in the required sequence and within the time allotted,' and entails managing one's time and adapting to time demands [17]. It is also known as daily time management [18].

People with cognitive impairments are known to have difficulties in managing their time for their daily activities [19-21]. Difficulties in time-related cognitive functions could present in different ways in different populations of older adults. Persons with dementia are known to have disorientation to the time, day and date, require more time for daily activities, difficulty in keeping track of past events, planning for future events, difficulty knowing, remembering and planning "when" and difficulty in judging the duration of different activities and situations [22]. Haj and Kapogiannis have described time distortions in dementia due to deficits in memory and time perception [23]. Difficulties in time estimation have been attributed to deficits in central executive function [24] and distortions in different components of the internal clock [25]. Requena-Komuro [26] have reported altered time awareness that contributes to abnormal behaviours in dementia syndromes such as clockwatching, reliving past events and temporal inflexibility and the Godot syndrome, i.e., anxiety symptoms regarding upcoming events by repeatedly asking questions. Difficulties with time estimation

For peer review only - http://bmjopen.bmj.com/site/about/guidelines.xhtml

have been reported in individuals with mild cognitive impairments [25, 27] and also in older adults without cognitive impairments [28].

Time-related deficits and associated behaviours affect the ability to do daily activities independently and add considerably to caregiver strain and distress. The ability to manage one's time effectively is an essential factor influencing a person's self-efficacy [29-30]. With increasing understanding of the importance of time-related abilities on performance of daily activities, several interventions are being investigated to help people with cognitive impairments orient and navigate time and do their daily activities. These include reality orientation [31], high-tech assistive devices such as time aids, automatic calendars [22, 32, 33], electronic devices [34], mobile-based applications [35], and low-tech assistive devices such as paper-based calendars [36], routine organizers [37] and training to improve organizational skills and habits [38-39].

The provision of interventions requires the use of sound assessments to screen, evaluate or identify the nature and severity of time-related cognitive deficits and their impact on performance of daily activities. They are also essential to monitor and document changes with intervention or disease progression. Assessments have also been used by occupational therapists to design interventions for children and youth having difficulties with time-related concepts [40, 41]. However, it is not clear how time-related cognitive functions and daily time management of older adults are evaluated in practice, what are the available assessments and how these assessments should be selected.

A preliminary search for existing reviews on this topic was done in the following databases: JBI database of Systematic Reviews and Implementation Reports, Cochrane Database of Systematic Reviews, Cumulative Index to Nursing and Allied Health Literature (CINAHL), PROSPERO, and PubMed. We did not find any review addressing assessment of

For peer review only - http://bmjopen.bmj.com/site/about/guidelines.xhtml

time-related concepts in older adults. The proposed scoping review intends to address this lacuna by systematically mapping research done in this area.

Research question

What are the available tools to assess time-related cognitive functions and daily time management in older adults?

Aim

The aim of this proposed scoping review is to investigate assessments tools for time-related cognitive functions and daily time management in older adults with and without cognitive impairments.

Objectives

- To map the assessments tools available for older adults with respect to time-related concepts.
- To describe the characteristics of available assessment tools including, purpose, type and format of the test, study populations, psychometric properties and feasibility.

METHODS

The protocol was drafted using the Joanna Briggs Institute (JBI) Reviewer's Manual [42]. The protocol is registered with the Open Science Framework (https://osf.io/4ptgy/). The review will be undertaken between August 2020 and March 2022 and will involve the following steps:

1. **Identification of relevant studies**: The selection criteria and search strategy to identify relevant studies are as follows:

   a. Selection criteria**:**

   - Articles with older adults as participants, i.e., people aged 60 years or older OR groups with an average age of 60 years or older [5, 43], with and without cognitive

For peer review only - http://bmjopen.bmj.com/site/about/guidelines.xhtml

For peer review only

impairments will be included. Articles that have older participants along with other age groups will also be included.

- Primary research articles (quantitative and mixed method studies) that discuss assessment tools for one or more time-related concepts described in the introduction, either as a separate/specific test or as a component of another test (such as a cognitive assessment), if results of that component are presented separately. This includes:
    - Pre-post, quasi-experimental and experimental (randomized and non-randomized controlled trials) designs evaluating the effectiveness of interventions targeting time-related concepts (if the outcome measures used include assessments of time-related concepts) and also observational studies investigating one or more time-related concepts. Articles with experimental laboratory-based designs will be excluded.
    - Validation studies, i.e., studies developing or validating an instrument or assessment tool that assesses one or more time-related concepts
- All peer-reviewed articles in English till date will be included. The language restriction to English is based on convenience and feasibility of the authors involved in the study group.

b. Search strategy: The key words will be identified and search strategy will be constructed with involvement of all the authors. The search will be conducted in the following databases, PubMed, CINAHL, SCOPUS, Web of Science, PsychINFO. First an initial limited search of at least two databases will be done such as PubMed and CINAHL. This will be followed by analysis of the text words contained in the title and abstract of the retrieved publications and the index terms used to describe the articles.

For peer review only - http://bmjopen.bmj.com/site/about/guidelines.xhtml

A second search using all identified keywords and index terms will be undertaken across all included databases. The reference list of included publications will be searched for additional sources. The bibliographic information of included publications will be combined and stored using EndNote. The PICO and keywords for the search are summarized in Table 1. See Supplementary material for an example. The search terms will be refined further if required. A combination of descriptors (e.g. MESH terms) and key words will be used with appropriate Boolean operators. The search strategy will be tailored to each database. We plan to conduct the search between September and October 2020. An updated search would be carried out later during the review period.

Table 1: Keywords for search

**PICO**	**Keywords**
Population:<br><br>Older adults with and without cognitive impairments	elderly, older, older age, aged, older adults, elderly persons, old, seniors, aging adults, older persons, geriatric, executive dysfunction, mild cognitive impairment, dementia, delirium, cognitive impairment, cognitive dysfunction, cognitive limitation, Alzheimer's, senile, psychiatric disorders, neurological disorders, stroke, Parkinson's,
Intervention:<br>Assessment tools	assessment tool, screening, tool, evaluation tool, diagnostic, psychometric properties, psychometrics, validity, reliability, test, prediction, predictive value, questionnaire, instrument,
Comparison:	---

For peer review only - http://bmjopen.bmj.com/site/about/guidelines.xhtml

Not applicable	
Outcome:<br><br>Time-related concepts	time, time sense, time perception, time orientation, time awareness, time processing abilities, time management/managing one´s time, routines, schedule, orientation, disorientation, time planning, executive functions, cognitive functions, cognition, cognitive abilities, judging time, time judgement, time organization, organizing time, task duration, activity duration, temporal, temporality, calendar.

For peer review only

2. **Selection of articles (relevance):** Two reviewers will independently review the title and abstract of the included identified publications against the selection criteria. They will be marked as 'potentially relevant' or 'potentially irrelevant'. Discrepancies, if any, will be resolved by discussion and consensus between the two reviewers. If not resolved, a third reviewer will be consulted. This will be pilot tested using the framework proposed by JBI [42]. Full text publications will be retrieved for studies regarded as 'potentially relevant', and will be reviewed independently and in duplicate for inclusion against the selection criteria. All reviewers will use a pilot-tested screening form developed for this review.

3. **Data extraction:** Two reviewers will independently review the included articles using a data extraction form. The data extraction form will be adapted from JBI [42]. It will be piloted for approximately five articles. Modifications if any will be made and the remaining articles will be reviewed with the finalized data extraction form. The details to be extracted would include:

For peer review only - http://bmjopen.bmj.com/site/about/guidelines.xhtml

a. Bibliometric data: title of the study, journal, year of publication, place (country) where the study was conducted.

b. Study details: aim/purpose/research question, study design, study setting (institution, community, in-patient, etc.), study population (older adults with cognitive impairment, without cognitive impairment, etc.), mean age of participants, sample size, recruitment strategy, etc.

c. Details of time-related assessments used in the study: time-related concepts being assessed, purpose/type of assessment (screening, diagnostic), language, format (computer/paper-pencil), mode of administration (self-report/therapist-administered; questionnaire/interview/observational), cost of using the assessment tool (paid/free), time for the test, psychometric properties, etc.

4. **Collating, summarizing and reporting the results**: An overview of the included publications will be provided in a tabular format to summarize the various aspects such as study type, aim, study design, settings, participants and time-related cognitive assessments used. As it is a scoping review, quality assessment of the identified articles will not be undertaken**.** The identified assessments will be mapped with regard to time-related concepts being assessed. A narrative synthesis will be carried out for test characteristics and to summarize the findings. Reporting will be done using the Preferred Reporting Items for Systematic reviews and Meta-Analyses extension for Scoping Reviews [44].

DISCUSSION

For peer review only - http://bmjopen.bmj.com/site/about/guidelines.xhtml

The present protocol outlines a scoping review of peer-reviewed articles on assessments available for time-related concepts in older adults and to our knowledge, this is the first of its kind. This review will advance knowledge about assessing time-related concepts in older adults. This area is gaining importance due to its impact on function and participation of older adults and their caregivers. The review will inform health professionals such as occupational therapists on the selection of appropriate instruments to evaluate different time-related concepts in older adults and guide clinical reasoning in planning interventions to support daily time management in older adults. This includes the provision of time management products. These products are in the top 50 priority Assistive Product List identified by WHO [45]. Older adults are expected to be major consumers of assistive products. In line with the mandate of United Nations Convention on the Rights of Persons with Disabilities (CRPD) member states are required to enable affordable access to these devices, making this a priority area in rehabilitation across countries. Global initiatives in this area would require the use of appropriate valid and reliable assessments as well as appropriate assistive devices that are less likely to be abandoned by older adults in need of support [46, 47]. Thus, provision of suitable products and adjunct interventions necessitates detailed evaluation of end users needs as suggested by the first global research, innovation, and education on assistive technology summit [48]. These assessments would also help to monitor and determine the effectiveness of time management products and objectively study the impact of these interventions on other variables such as caregiver burden, quality of life, well-being, occupational participation, etc. Finally, the review will identify gaps to be addressed in future research. If sufficient literature is identified, a future systematic review or meta-analysis may be warranted.

Ethics and dissemination

For peer review only - http://bmjopen.bmj.com/site/about/guidelines.xhtml

As a secondary analysis, ethics approval is not required for this scoping review. We plan to disseminate the results through peer-reviewed journals and conferences targeting health professionals working with older adults. Amendments to the protocol, if any, will be stated in any future publication based on the review presented in this protocol.

Author Affiliations

[1]Department of Occupational Therapy and Centre for Studies on Healthy Aging, Manipal College of Health Professions, Manipal Academy of Higher Education, Manipal, Karnataka, India

[2]Meena Ramachandran, Faculty of Health Sciences, McMaster University, Canada

[3]Yuko Nishiura, Research Institute, National Rehabilitation Center for Persons with Disabilities, Department of Assistive Technology, Japan

[4]Bhumika Tumkur Venkatesh, Public Health Evidence South Asia, Prasanna School of Public Health, Manipal Academy of Higher Education, Manipal, Karnataka, India

[5]Lena Dahlberg, School of Health and Welfare, Dalarna University, Sweden; and Aging Research Center, Karolinska Institutet & Stockholm University, Sweden

Acknowledgements: The authors would like to thank Dr. Gunnel Janeslätt, Researcher, Center for Clinical Research, Dalarna University, Dalarna and associated to Centre for Public Health and Caring Sciences, Disability and Habilitation, Uppsala University, Uppsala, Sweden for her valuable guidance and support to this scoping review protocol.

Contributors: All authors have made substantive intellectual contributions to the development of this protocol. SAD conceived the idea of this research, followed by

For peer review only - http://bmjopen.bmj.com/site/about/guidelines.xhtml

discussions with the other authors that contributed to finalising the research idea. All authors worked on the methodology of the scoping review. SAD drafted the protocol with inputs from MR, YN and BTV which was further reviewed and revised by LD.

Funding: This scoping review is part of an ongoing international research collaboration “Managing Time in Dementia" funded by the Indian Council of Medical Research (54/1/GER/Indo-Sweden/17-NCD-II), India, FORTE, (FORTE 2017-00029) Sweden and Japan Society for the Promotion of Science, Grant-in-Aid for Young Scientists (B) [16K16458], Japan.

Competing interests: None declared.

Patient and public involvement: Patients and/or the public were not involved in the design, or conduct, or reporting, or dissemination plans of this research.

Patient consent for publication: Not required.

References

1. World Health Organization. Ageing and Health, 2018. Available at: https://www.who.int/news-room/fact-sheets/detail/ageing-and-health (accessed 29 September 2020).

2. United Nations. World Population Ageing Highlights, 2017. Available at: https://www.un.org/en/development/desa/population/publications/pdf/ageing/WPA2017_Highlights.pdf (accessed 29 September 2020).

For peer review only - http://bmjopen.bmj.com/site/about/guidelines.xhtml

3. GBD 2016 Neurology Collaborators. Global, regional, and national burden of neurological disorders, 1990–2016: a systematic analysis for the Global Burden of Disease Study 2016. *Lancet Neurol* 2019;18:459–80. doi:https://doi.org/10.1016/S1474-4422(18)30499-X

4. Global Burden of Disease Collaborative Network, 2012. Global Burden of Disease Study 2010 (GBD 2010) Results by Cause 1990-2010. Seattle, United States: Institute for Health Metrics and Evaluation (IHME). doi:https://doi.org/10.6069/J86D-DZ43

5. World Health Organization. The epidemiology and impact of dementia: current state and future trends, 2015. Available at https://www.who.int/mental_health/neurology/dementia/dementia_thematicbrief_epidemiology.pdf (accessed 29 September 2020).

6. World Health Organization. Dementia Fact Sheet, 2020. Available at: www.who.int/mediacentre/factsheets/fs362/en/ (accessed 29 September 2020).

7. Mitchell AJ, Kemp S, Benito-León J, Reuber M. The influence of cognitive impairment on health-related quality of life in neurological disease. *Acta Neuropsychiatr* 2010;22:2-13 doi:10.1111/j.1601-5215.2009.00439.x

8. Danovska M, Stamenov B, Alexandrova M, Peychinska D. Post-stroke cognitive impairment - phenomenology and prognostic factors. *Journal of IMAB* 2012;18:290-297. doi:10.5272/jimab.2012183.290

9. Cullen B, Smith DJ, Deary IJ, et al. The ‘cognitive footprint’ of psychiatric and neurological conditions: cross-sectional study in the UK Biobank cohort. *Acta Psychiatr Scand* 2017;135:593-605. doi:10.1111/acps.1273

For peer review only

For peer review only - http://bmjopen.bmj.com/site/about/guidelines.xhtml

10. Rotenberg S, Maeir A, Dawson DR. Changes in activity participation among older adults with subjective cognitive decline or objective cognitive deficits. *Front Neurol* 2020;10:1393. doi:10.3389/fneur.2019.01393

11. Black CM, Ritchie CW, Khandker RK et al. Nonprofessional caregiver burden is associated with the severity of patients' cognitive impairment. *PLoS One* 2018;13:e0204110. doi:https://doi.org/10.1371/journal.pone.0204110

12. Rosenblum S, Regev N. Timing abilities among children with developmental coordination disorders (DCD) in comparison to children with typical development. *Res Dev Disabil* 2013;34:218-227. doi:https://doi.org/10.1016/j.ridd.2012.07.011

13. World Health Organization. International Classification of Functioning Disability and Health, 2001. Available at https://www.who.int/standards/classifications/international-classification-of-functioning-disability-and-health (accessed 4 December 2020)

14. Janeslätt G. Validity in assessing time processing ability, test equating of KaTid-Child and KaTid-Youth. *Child Care Health Dev* 2012;38:371-378. doi:https://doi.org/10.1111/j.1365-2214.2011.01249.x

15. Janeslätt G, Granlund M, Alderman I et al. Development of a new assessment of time processing ability for children, using Rasch analysis. *Child Care Health Dev* 2008;34:771-780. doi:10.1111/j.1365-2214.2008.00865.x

16. Janeslätt G, Kottorp A, Granlund M. Evaluating intervention with time aids in children with disabilities. *Scand J Occup Ther* 2014;21:181-190. doi:https://doi.org/10.3109/11038128.2013.870225

17. WHO. ICF Update Platform, 2021. Available from: https://extranet.who.int/icfrevision/nr/loginICF.aspx. (accessed 11 January, 2021)

For peer review only - http://bmjopen.bmj.com/site/about/guidelines.xhtml

18. Janeslätt G, Lindstedt H, Adolfsson P. Daily time management and influence of environmental factors on use of electronic planning devices in adults with mental disability. *Disabil Rehabil Assist Technol* 2015;10:371–377.

19. Davies D, Stock S, Wehmeyer M. Enhancing independent time-management skills of individuals with mental retardation using a palmtop personal computer. *Ment Retard* 2002;40:358-65. doi:10.1352/0047-6765(2002)040<0358:EITMSO>2.0.CO;2.

20. Toner M, O'Donoghue T, Houghton S. Living in chaos and striving for control: How adults with Attention Deficit Hyperactivity Disorder deal with their disorder. *Intl J Disabil Dev Educ* 2006;53:247-261. doi: 10.1080/10349120600716190

21. Borson S. Cognition, Aging and Disabilities: Conceptual Issues. *Phys Med Rehabil Clin N Am* 2010;21:375-382. doi:10.1016/j.pmr.2010.01.001

22. Nygård L, Johannson M. The experience and management of temporality in five cases of dementia. *Scand J Occup Ther* 2001;8:85-95. doi:https://doi.org/10.1080/11038120120542

23. Haj EM, Kapogiannis D. Time distortions in Alzheimer's disease: a systematic review and theoretical integration. *NPJ Aging Mech Dis* 2016; 2:1-5. doi:10.1038/npjamd.2016.16;

24. Papagno C, Allegra A, Cardaci M. Time estimation in Alzheimer's disease and the role of the central executive. *Brain and Cognition* 2004;54:18-23. doi:10.1016/S0278-2626(03)00237-9

25. Caselli L, Iaboli L, Nicheli P. Time estimation in Alzheimer's disease patients. *Behav Brain Sci* 2009;5:1-10. doi:10.1186/1744-9081-5-32

26. Requena-Komuro MC, Marshall CR, Bond RL, et al. Altered Time Awareness in Dementia. *Front Neurol* 2020;11:1-12. doi:10.3389/fneur.2020.00291

For peer review only

For peer review only - http://bmjopen.bmj.com/site/about/guidelines.xhtml

27. Rueda AD, Schmitter-Edgecombe M. Time estimation abilities in mild cognitive impairment and Alzheimer's disease. *Neuropsychology* 2009;23(2):178–188. doi:10.1037/a0014289

28. Toglia J, Lahav O, Ari EB, et al. Adult age and cultural differences in performance on the Weekly Calendar Planning Activity (WCPA). *Am J Occup Ther* 2017;71(5):7105270010p1-8. doi:10.5014/ajot.2016.020073

29. Bembenutty H. Academic delay of gratification, self-efficacy, and time management among academically unprepared college students. *Psychol Rep* 2009;104:613-23. doi:10.2466/pr0.104.2.613-623

30. Hayat RA, Janeslätt G, White S et al. Time management skills in relation to general self-efficacy and parental sense of competence in individuals with and without cognitive disabilities. *Cogent Psychol 2019*;6:1. doi: 10.1080/23311908.2019.1655981

31. Camargo, CHF, Justus FF, Retzlaff G. The effectiveness of reality orientation in the treatment of Alzheimer's Disease. *Am J Alzheimers Dis Other Demen* 2015;30:527-532. doi: 10.1177/1533317514568004

32. Lindqvist E, Nygard L, Borrell L. Significant junctures on the way towards becoming a user of assistive technology in Alzheimer's disease. *Scand J Occup Ther* 2013;20:386-396. doi: 10.3109/11038128.2013.766761

33. Nishiura Y, Nihei M, Nakamura-Thomas H, et al. Effectiveness of using assistive technology for time orientation and memory, in older adults with or without dementia. *Disabil Rehabil Assist Technol* 2019;19:1-7. doi:10.1080/17483107.2019.1650299

34. Janeslätt G, Lindstedt H, Adolfsson P. Daily time management and influence of environmental factors on use of electronic planning devices in adults with mental disability. *Disabil Rehabil Assist Technol* 2014;10:371-377. doi: 10.3109/17483107.2014.917124.

For peer review only - http://bmjopen.bmj.com/site/about/guidelines.xhtml

35. Jamieson M, Cullen B, Lennon M, et al. Designing ApplTree: usable scheduling software for people with cognitive impairments. *Disabil Rehabil Assist Technol* 2020. doi:10.1080/17483107.2020.1785560.

36. Greenaway MC, Duncan NL, Smith GE. The memory support system for mild cognitive impairment: randomized trial of a cognitive rehabilitation intervention. *Int J Geriatr Psychiatry* 2013;28:402-409.

37. Assis LDO, Tirado MGA, Pertence AEDM, et al. Evaluation of cognitive technologies in geriatric rehabilitation: A case study pilot project. *Occup Ther Int* 2010;17:53–63.

38. Holmefur M, Lidström-Holmqvist K, Roshanay AH, et al. Pilot study of Let's Get Organized: A group intervention for improving time management. *Am J Occup Ther* 2019;73:7305205020. Available at: https://doi.org/10.5014/ajot.2019.032631

39. White S. Let's get organized: An intervention for persons with co-occurring disorders. *Psychiatr Serv* 2007 May;58(5). Available at: https://ps.psychiatryonline.org/doi/full/10.1176/ps.2007.58.5.713 (accessed 29 September 2020).

40. Wennberg B, Janeslätt G, Kjellberg A, et al. Effectiveness of time-related interventions in children with ADHD aged 9-15 years: a randomized controlled study. *Eur Child Adolesc Psychiatry* 2018;27:329-342. doi: 10.1007/s00787-017-1052-5.

41. Janeslätt G, Granlund M, Kottorp A. Measurement of time processing ability and daily time management in children with disabilities. *Disabil Health J* 2009;2:15-9. doi: 10.1016/j.dhjo.2008.09.002.

For peer review only

For peer review only - http://bmjopen.bmj.com/site/about/guidelines.xhtml

42. Peters MDJ, Godfrey C, McInemey P, et al. Scoping reviews. In: Aromataris E, Munn Z, eds. Joanna Briggs Institute Reviewer's Manual. Adelaide, Australia: Joanna Briggs Institute; 2020:407-454.

43. Dahlberg L, Ahlström G, Bertilsson G, et al. (2019). Kunskapsläget för bedömning och insatser inom äldreomsorgen [Social care and services for older adults. An evidence map addressing assessment and interventions]. Report 306/2019. Stockholm: Swedish Agency for Health Technology Assessment and Assessment of Social Services (SBU). Available at: https://www.sbu.se/sv/publikationer/sbu-kartlagger/kunskapslaget-for-bedomning-och-insatser-inom-aldreomsorgen2/ (accessed 18 August, 2021)

44. Tricco AC, Lillie E, Zarin W, et al. PRISMA extension for scoping reviews (PRISMA-ScR): checklist and explanation. *Ann Intern Med* 2018;169:467-473. doi:10.7326/M18-0850.

45. World Health Organization. Priority Assistive Products, 2016. Available at. https://www.who.int/phi/implementation/assistive_technology/low_res_english.pdf. (accessed 12 August, 2021)

46. Demers L, Fuhrer MJ, Jutai JW, et al. Tracking mobility-related assistive technology in an outcomes study. *Assist Technol* 2008;20(2):73–83. doi: 10.1080/10400435.2008.10131934.

47. Phillips B, Zhao H. Predictors of assistive technology abandonment. *Assist Technol* 1993;5(1):36–45. doi: 10.1080/10400435.1993.10132205.

48. Smith RO, Scherer MJ, Cooper R, et al. Assistive technology products: a position paper from the first global research, innovation, and education on assistive technology (GREAT) summit. *Disabil Rehabilitation Assist Technol* 2018 13:5, 473-485, doi: 10.1080/17483107.2018.1473895

For peer review only - http://bmjopen.bmj.com/site/about/guidelines.xhtml

Assessment of time-related deficits in older adults: A scoping review protocol

Supplementary material. Example of search strategy for PubMed

(("SCREENING"[Title/Abstract] OR "ASSESSMENT"[Title/Abstract] OR "assess*"[All Fields] OR "TOOL"[Title/Abstract] OR "SCALE"[Title/Abstract] OR "INSTRUMENT"[Title/Abstract] OR "MONITOR"[Title/Abstract] OR "EVALUATE"[Title/Abstract] OR "IDENTIFY"[Title/Abstract] OR "TEST"[Title/Abstract]) AND ("TIME RELATED"[Title/Abstract] OR "TIME ORIENTATION"[Title/Abstract] OR "ROUTINE ACTIVITIES"[Title/Abstract] OR "COGNITIVE DEFICIT"[Title/Abstract]) AND ("humans"[MeSH Terms] AND 2000/01/01:2020/09/20[Date - Publication] AND ("middle aged"[MeSH Terms] OR "aged"[MeSH Terms]))) AND ((humans[Filter]) AND (2000/1/1:2020/9/20[pdat]) AND (middleaged[Filter] OR aged[Filter]))

For peer review only
